# Persistent postural-perceptual dizziness: a phenotype-oriented approach to practical management

**DOI:** 10.3389/fneur.2026.1879813

**Published:** 2026-08-03

**Authors:** Mar Rey-Berenguel, Javier Vallecillo-Zorrilla, Alicia Jimenez-Campos, Juan Manuel Espinosa-Sanchez

**Affiliations:** 1 Division of Otoneurology, Department of Otolaryngology, Hospital Universitario Virgen de las Nieves, Granada, Spain; 2 Division of Otolaryngology, Department of Surgery, University of Granada, Granada, Spain; 3 Otology and Neurotology Group CTS495, Instituto de Investigación Biosanitaria ibs.GRANADA, Granada, Spain

**Keywords:** chronic dizziness, functional vestibular disorder, migraine overlap, multimodal management, persistent postural-perceptual dizziness, phenotyping, vestibular rehabilitation, visual dependence

## Abstract

**Introduction:**

Persistent postural-perceptual dizziness (PPPD) is a chronic functional vestibular disorder defined by persistent dizziness, unsteadiness, or non-spinning vertigo exacerbated by upright posture, active or passive motion, and visually complex environments. In clinical practice, however, the diagnostic label alone is often insufficient to guide management, because patients differ substantially in the relative prominence of visual dependence, motion–postural burden, multisensory provocation, affective-hypervigilant mechanisms, and migraine overlap.

**Objective:**

To propose a clinically oriented, dimension-informed framework for translating a positive diagnosis of PPPD into individualized formulation and practical multimodal management.

**Methods:**

This article presents a practice-oriented narrative synthesis rather than a systematic review or formal guideline. The literature was selected to inform clinical formulation, symptom profiling, assessment, treatment prioritization, and the interface between PPPD and vestibular migraine. The proposed framework is intended as a pragmatic clinical heuristic, not as a biologic taxonomy or a validated treatment-stratification model.

**Conclusion:**

We organize PPPD heterogeneity around five anchor clinical phenotypes: visually dominant/visually dependent, active motion–postural, mixed/multisensory, affective-hypervigilant, and migraine-overlap/vestibular migraine–PPPD interface. These patterns are best understood as dominant clinical dimensions within a continuous and overlapping clinical space rather than as mutually exclusive phenotypes. This dimensional formulation may guide the weighting and sequencing of vestibular rehabilitation, psychologically informed interventions, migraine-oriented management, and selected pharmacologic treatment. It may also support the rational selection of assessment tools for clinical formulation and longitudinal follow-up. Prospective studies are needed to determine whether these clinical dimensions predict prognosis, functional outcome, treatment needs, or differential treatment response.

## 1 Introduction

Persistent postural-perceptual dizziness (PPPD) is a chronic functional vestibular disorder characterized by persistent dizziness, unsteadiness, or non-spinning vertigo that is exacerbated by upright posture, active or passive motion, and exposure to moving or complex visual environments (1). The Bárány Society criteria represented an important conceptual advance by replacing a previously fragmented nomenclature (including phobic postural vertigo, chronic subjective dizziness, visual vertigo, and related labels) with a single positive diagnostic construct (1, 2). This shift remains clinically important because it clarifies that PPPD is neither a residual label applied after unrevealing testing nor a purely psychiatric explanation for chronic dizziness, but a recognizable disorder with a characteristic symptom architecture, compatible precipitants, and meaningful functional consequences (1, 3). At the same time, PPPD commonly emerges after vestibular disorders, migraine-related events, medical illness, or psychological distress, so establishing the diagnosis, although essential, does not by itself complete the clinical task (1, 4).

Once PPPD has been diagnosed, patients do not all present in the same clinical way. Some are dominated by visual complexity and visually moving scenes; others by gait, turning, posture, and self-motion; others by broader multisensory amplification; and others still by hypervigilance and avoidance (3, 5). Neuroimaging studies further support these patterns as expressions of a network-level functional substrate, with altered connectivity among multimodal vestibular, visual-motion, spatial-cognitive, and affective-attentional regions (6–9). In some patients, the persistent influence of migraine-related mechanisms further shapes the clinical picture (10). This internal heterogeneity is not a marginal issue: it shapes disability, the patient’s explanatory model, treatment engagement, the relative relevance of vestibular rehabilitation, and the clinical weight of psychological or migraine-oriented interventions (2, 11, 12). For that reason, a purely syndromic description of PPPD is often insufficient for practical management. What the clinician still needs is a second layer of formulation that clarifies which symptom-generating and symptom-maintaining processes are most clinically dominant in the individual case (3, 4).

Current evidence supports recurring domains of heterogeneity and some reproducible clinical patterns within PPPD, but it does not justify a rigid internal taxonomy, mutually exclusive disease classes, or validated treatment strata (5, 13). For this reason, the phenotype-oriented framework developed in this review is offered as a clinically useful heuristic for formulation and prioritization (not as a biologically grounded subtype system, nor as an empirically validated stratification model) and is intended to translate heterogeneity into more explicit clinical reasoning without overstating the evidence base.

The highest-level evidence syntheses currently available do not support strong, treatment-specific recommendations in PPPD. The 2023 Cochrane review of pharmacological interventions identified no placebo-controlled randomized trials of selective serotonin reuptake inhibitors (SSRIs), serotonin–norepinephrine reuptake inhibitors (SNRIs) or other pharmacological agents meeting Bárány Society diagnostic criteria (14), and the companion 2023 Cochrane review of non-pharmacological interventions likewise found no eligible randomized trial evidence sufficient to support any “gold standard” treatment for PPPD (15). Post-Cochrane systematic reviews and meta-analyses of vestibular rehabilitation, vestibular physical therapy, conservative treatment, and adjunctive cognitive-behavioral approaches have reported clinically meaningful improvements in dizziness-related handicap, but with substantial between-study heterogeneity, variable comparators, differing follow-up durations, and inconsistent outcome selection (16–20). Far from invalidating a phenotype-oriented approach, this evidence landscape is precisely what makes such an approach necessary: in the absence of a high-quality, hierarchized evidence base capable of stratifying treatment by validated subtype, clinicians still need a disciplined, clinically grounded way to prioritize and integrate available modalities in the individual case.

The aim of this review is therefore to organize diagnosis, formulation, assessment, and multimodal management around clinically recognizable anchor phenotypes within a dimensional formulation framework, offered as a practical framework for the intermediate clinical step between diagnosis and treatment selection in everyday neuro-otology care (2, 3).

## 2 Scope of the review and literature approach

This article was developed as a practice-oriented narrative synthesis rather than as a systematic review, exhaustive evidence map, or formal guideline. The format was chosen to address a practical question that is not fully answered by diagnostic criteria alone: how a single positive diagnosis can be translated into explicit clinical formulation and individualized multimodal management. The manuscript therefore does not aim to provide a comprehensive or hierarchized appraisal of all available PPPD literature. This approach allows selected clinically relevant evidence to be synthesized around recurring domains of heterogeneity, maintenance mechanisms, assessment tools, and treatment priorities.

The literature search was conducted in PubMed/MEDLINE, Scopus, and Embase, with supplementary cross-referencing of relevant bibliographies, covering publications from January 2017, when the Bárány Society diagnostic criteria were formally established, through 10 April 2026. Search terms combined “persistent postural-perceptual dizziness” and “PPPD” with modifiers related to diagnosis, phenotypes, subtypes, heterogeneity, clinical assessment, vestibular rehabilitation, cognitive-behavioral therapy, pharmacotherapy, vestibular migraine, and relevant assessment instruments. Publications in English were considered. Selected pre-2017 works were retained when they provided conceptual foundations for PPPD and its precursor constructs (phobic postural vertigo, chronic subjective dizziness, visual vertigo, space-motion discomfort). The literature approach prioritized consensus documents, systematic reviews and meta-analyses, clinically oriented and treatment-focused reviews, clinical trials and interventional studies, studies addressing precipitating conditions, comorbidities, and maintenance mechanisms, and exploratory work on internal heterogeneity and symptom subtypes. Validation studies of instruments relevant to PPPD symptom profiling and follow-up were also prioritized because they are directly relevant to phenotype-oriented practice. Where direct PPPD evidence was limited, indirect evidence was incorporated only when it clarified clinical formulation, disability, quality of life, or multimodal management in ways that were practically relevant to bedside care and did not require disproportionate inference.

Accordingly, the purpose of this literature approach was not exhaustive evidence mapping, but transparent, practice-oriented synthesis. No PRISMA-based screening, predefined inclusion algorithm, or formal risk-of-bias appraisal was undertaken, and the present work should therefore be read as a practice-oriented narrative synthesis rather than as a systematic evidence evaluation. The review was designed to connect positive diagnosis, phenotype-oriented formulation, practical assessment, and treatment prioritization in a way that remains clinically useful, methodologically proportionate, and applicable to real-world neuro-otology practice.

## 3 Diagnostic framework before phenotyping

### 3.1 Positive clinical diagnosis of PPPD

Before moving to phenotyping, it is essential to establish PPPD as a positive clinical diagnosis rather than as a residual label for patients with persistent dizziness and unrevealing testing. The Bárány Society criteria define PPPD by one or more symptoms of dizziness, unsteadiness, or non-spinning vertigo present on most days for at least 3 months, exacerbated by upright posture, active or passive motion, and exposure to moving or complex visual stimuli, and precipitated by conditions that cause vertigo, unsteadiness, dizziness, or relevant psychological distress (1). This formulation is clinically important because it grounds diagnosis in a recognizable symptom architecture and compatible temporal course rather than in the mere absence of alternative structural explanations (1, 2).

In practice, diagnosis begins with careful clarification of what the patient means by “dizziness.” In PPPD, dominant complaints usually involve persistent unsteadiness, disturbed spatial orientation, or non-spinning motion sensations rather than recurrent brief spinning attacks or isolated presyncope (1, 2). Symptoms are typically present on most days, often fluctuating in intensity rather than disappearing completely, and are embedded in a characteristic pattern of worsening during walking, standing, head or body motion, and visually complex environments (1, 4). For that reason, the clinical history should establish not only chronicity, but also the triad of posture-related, motion-related, and visually induced exacerbation that gives PPPD its diagnostic coherence (1, 21).

A compatible precipitating context is equally important. PPPD may follow acute vestibular events (e.g., vestibular neuritis), episodic vestibular syndromes (e.g., vestibular migraine [VM] or benign paroxysmal positional vertigo [BPPV]), chronic vestibular disorders, other medical or neurologic illnesses, or periods of marked psychological distress (1).

PPPD is treated throughout this review as a positive clinical diagnosis defined by the Bárány Society criteria, which are explicitly phenomenological (1). The mechanistic constructs used later in this manuscript (including visual dependence, maladaptive sensory reweighting, body vigilance, and threat-related processing) refer to currently proposed maintenance mechanisms that inform clinical formulation and rehabilitation design but are not themselves diagnostic criteria, and their presence or absence in a given patient does not modify whether PPPD is formally diagnosable. This distinction matters because phenotype-oriented reasoning, as developed below, operates at the level of formulation rather than at the level of diagnostic definition.

### 3.2 Differential diagnosis, overlap syndromes, and active comorbid drivers

The differential diagnosis of PPPD is less about identifying a single rival disorder than about determining which conditions remain active, which have become residual, and which now coexist with the chronic syndrome (1, 3). A common clinical error is to attribute all chronic symptoms to a previous vestibular event without asking whether the original disorder has resolved and a persistent functional vestibular disorder has emerged on top of it (2, 3). This issue is especially relevant after vestibular neuritis, BPPV, Meniere disease, or VM, where episodic symptoms may coexist with continuous PPPD-related background dizziness (3, 10).

VM deserves particular attention because it is one of the most frequent overlap zones in practice (4, 10). The key distinction is temporal and syndromic rather than purely phenomenological. VM remains primarily an episodic vestibular disorder, even though some patients present with prolonged or clinically more chronic migrainous vestibular symptoms; PPPD, by contrast, is defined by persistent symptoms on most days with posture-, motion-, and visual-dependence features beyond or between attacks (1, 10). At the same time, VM and PPPD should be understood as potentially overlapping or coexisting disorders; whether they also sit along a broader episodic-to-chronic vestibular spectrum remains a useful conceptual framing rather than a settled classificatory conclusion (10). Such overlap should prompt formulation rather than diagnostic paralysis.

Residual vestibular abnormalities may also complicate assessment without negating the diagnosis. Clinical studies have reported unilateral vestibular hypofunction, otolith abnormalities, and isolated otolith dysfunction in subsets of patients meeting PPPD criteria (4, 22). Such findings may modulate symptom expression, but they do not in themselves explain the full chronic syndrome once maladaptive perceptual weighting, avoidance, and vigilance have become established (3). In this sense, residual findings are better understood as active or residual drivers within the formulation than as automatic refutations of the diagnosis.

A clinically important caveat concerns atypical or multicanal BPPV, including anterior semicircular canal involvement, which may produce predominantly non-spinning dizziness rather than clear positional vertigo and can therefore be mistaken for a migrainous or functional disorder. Because such presentations are potentially treatable with appropriate repositioning, they warrant active exclusion before a persistent functional label is accepted, particularly in patients previously diagnosed with vestibular migraine and unresponsive to serotonergic medication. A structured reappraisal of dizziness-handicap responses (23) and targeted positional testing, including maneuvers proposed for anterior-canal and multicanal disease (24), may help identify treatable canal pathology in this setting.

Anxiety, panic, and hypervigilance require equally careful handling. They may precede PPPD, precipitate it, emerge as consequences of it, or coexist with it as active amplifiers of disability (2, 4). The diagnostic mistake here is to collapse the whole syndrome into “psychogenic dizziness” once panic symptoms or health anxiety are identified (2). A more accurate reading is often that PPPD and anxiety-related processes interact bidirectionally, with autonomic arousal, fearful expectation, body vigilance, and avoidance contributing to persistence after a vestibular or other trigger (3, 13).

Orthostatic and autonomic disorders also deserve consideration in patients whose symptoms are strongly linked to standing or walking. PPPD and orthostatic dizziness may overlap at the level of posture-related worsening, but they differ in symptom quality, associated physiology, and the role of visual complexity and motion provocation (25). Presyncope, postural tachycardia, or hemodynamic triggers should therefore be actively sought when the history suggests them, especially when the dizziness is not clearly multisensory or visually modulated (25). More broadly, the diagnostic task in PPPD is not to purge the case of comorbidity, but to identify which overlap syndromes and active drivers remain clinically relevant and therefore require parallel management (1, 3).

### 3.3 From diagnosis to clinical formulation

Once PPPD has been positively diagnosed, the next step is not indefinite investigation, but translation of the case into a functional clinical formulation. Diagnosis identifies the disorder; formulation identifies what is currently driving it (2, 3). A syndromic label alone does not tell the clinician whether visual dependence, self-motion sensitivity, threat monitoring, migraine overlap, residual vestibular dysfunction, or broader multisensory amplification should be given greatest practical weight (3, 5).

## 4 From heterogeneity to clinical phenotyping

### 4.1 What current evidence supports: domains, clusters, and limitations

PPPD is diagnostically unified, yet clinically heterogeneous in how its core symptom architecture is expressed. The Bárány Society criteria intentionally consolidated a previously fragmented field that had been described under labels such as phobic postural vertigo, space-motion discomfort, visual vertigo, and chronic subjective dizziness (1). That historical convergence remains clinically relevant because it helps explain why PPPD functions as a single syndromic construct while still displaying substantial internal variation in symptom weighting, perceptual style, illness behavior, and affective loading (1, 2).

The clearest empirical attempt to organize this variation comes from Yagi et al., who used factor and cluster analysis of the Niigata PPPD Questionnaire to identify three recurring patterns: visual-dominant, active motion-dominant, and mixed (5). These findings suggest that clinical variability in PPPD is not merely anecdotal, but shows some reproducible internal organization. More specifically, symptom aggravation appears to organize around differences in the relative predominance of core provoking factors, with visual triggers accounting for the largest proportion of variance (5).

Even so, these data should not be over-interpreted. In Yagi’s framework, patients meeting PPPD criteria were still expected to show aggravation across all canonical domains, with clustering reflecting relative weighting rather than genuinely discrete disease classes (5). The broader literature is methodologically diverse and behaves in the same way: it supports the language of recurring clinical patterns more convincingly than any finalized internal taxonomy (3, 11, 13).

Neuroimaging findings provide a complementary, although still non-diagnostic, layer of support for a dimensional view of PPPD. Resting-state and task-based functional MRI studies have implicated altered coupling among multimodal vestibular cortical regions, insular and opercular cortices, occipito-parietal visual-motion areas, spatial-cognitive networks, prefrontal regulatory regions, and cerebellar circuits (6–9). In particular, patients with PPPD have shown reduced connectivity within networks involved in multisensory vestibular processing and spatial cognition, together with increased coupling between visual and emotional-processing networks (6). More recent work has further suggested that visual stimulation may reveal abnormal vestibulo-visuo-somatosensory reweighting, with reduced vestibular–visual connectivity, increased somatosensory–visual coupling, and enhanced connectivity from visual areas toward spatial-cognitive and prefrontal regions (7). Graph-theoretical analyses also indicate less efficient local and global information transfer, with abnormal nodes in visual, sensorimotor, multisensory vestibular, precuneus, and cerebellar regions (8). These observations are compatible with the visually dominant, active motion–postural, and mixed/multisensory phenotypes, and they also support the relevance of affective-attentional mechanisms in some patients. However, current neuroimaging evidence should not be interpreted as defining biologically discrete PPPD subtypes. Rather, it supports the concept that clinically recognizable phenotypes may reflect different relative weights within a shared visuo-vestibulo-somatosensory and affective-attentional network dysfunction.

### 4.2 Three practical axes of phenotype-oriented formulation

If this clinical variability is to become useful at the bedside, it must be translated into a limited set of formulation axes. The first axis is the relative predominance of exacerbating domains. This axis captures whether the dominant aggravation arises from visually complex environments, active self-motion, passive motion, upright posture, or a more evenly distributed combination of these. It is the axis most directly informed by both the symptom architecture embedded in the Bárány criteria and the clustering data reported by Staab et al. (1) and Yagi et al. (5).

The second axis is the degree of visual dependence. This refers not simply to whether visual aggravation is present, but to the extent to which visually complex or visually moving environments dominate the patient’s illness experience. This distinction matters clinically because PPPD is associated with greater visual vertigo than VM across a range of ecologically relevant situations (26). In some patients, visual dependence is therefore not merely one symptom among others, but a central organizing feature of the disorder (26). Emerging perceptual data add a useful perceptual and potentially mechanistic nuance to this axis. In a controlled motion-perception study, patients with PPPD required a higher proportion of coherent visual motion than healthy controls to identify motion direction, and that threshold increased with depressive burden (27). This suggests that visually dominant presentations may involve not only symptom provocation by visual environments, but also altered processing of complex visual motion under conditions of uncertainty (27).

The third axis is affective burden and anxiety-related amplification. PPPD is not reducible to anxiety, but anxiety-related mechanisms, balance vigilance, negative cognitive appraisal, and maladaptive illness beliefs may act as contributing factors to chronicity, symptom amplification, and disability, and function as predictors or mediators of clinically relevant symptom burden in several cohorts (3, 13, 28). Contemporary explanatory models of PPPD persistence, rather than of PPPD diagnosis, emphasize pre-existing anxiety vulnerability, body vigilance, illness-related threat processing, and negative illness beliefs as mechanisms that may contribute to chronicity and amplification, without these mechanisms being required for the disorder to be formally diagnosed (3, 28). In practice, this axis helps distinguish patients in whom anxiety is simply present from those in whom hypervigilance, anticipatory threat, and self-monitoring function as major maintenance mechanisms (3, 13). These three axes describe the current clinical state of the patient; additional cross-cutting variables further refine how that state is expressed, and are considered next.

### 4.3 Cross-cutting modifiers: age, precipitant context, chronicity, and comorbidity structure

Some clinically relevant variables are better understood as cross-cutting modifiers than as phenotypes in their own right. Age is one such modifier. Zhang et al. reported fewer emotional disorders and lower dizziness-related handicap in older patients with PPPD, whereas Yagi et al. found that the active motion-dominant group was older than the visual-dominant group (5, 29). Consistent with this, Fukushima et al. reported age-related differences in PPPD, with younger patients showing higher anxiety scores and older patients showing a higher proportion of peripheral vestibular diseases among precipitating conditions (30). Taken together, these observations suggest that age may modulate phenotype expression, particularly affective loading, precipitant context, and the balance between motion-related and visually dominated symptoms, without justifying an age-defined phenotype (5, 29, 30).

Precipitant context is a further relevant modifier. By definition, PPPD follows a compatible trigger, yet the nature of that trigger still matters for clinical formulation without defining a separate phenotype in itself (1). It may emerge after acute or episodic vestibular events, chronic vestibular disorders, other medical or neurologic conditions, or periods of marked psychological distress (1). Common associated or precipitating diagnoses include VM and anxiety disorders, while residual vestibular abnormalities may remain identifiable in a substantial subset of patients (4). Waterston et al. emphasized that precipitating events and comorbidity structure are not merely background features but part of the clinical context that helps explain why apparently similar PPPD diagnoses may differ in dominant symptom patterns and management priorities (4). Likewise, the distinction between primary and secondary chronic functional dizziness suggests that trigger context may shape presentation without determining a separate final disorder in categorical terms (31).

Chronicity is a further cross-cutting modifier. Longer symptom duration does not generate a new phenotype by itself, but it may be associated with consolidation of bodily vigilance, fear of symptoms, avoidance, and disability (1, 12, 13, 32). This association should be interpreted cautiously, because most of the available data are cross-sectional or retrospective and the apparent effect of duration is likely confounded by variables that frequently covary with chronicity. Within the PPPD literature itself, baseline symptom severity, pre-existing psychological vulnerability, and the precipitant context of the original event are documented to vary substantially across patients in ways that may inflate the apparent independent effect of duration on disability (13, 31). Standard methodological caveats of observational research also apply, including prior access and adherence to treatment and retrospective recruitment bias toward more severely affected patients in tertiary clinical series. In the absence of longitudinal controlled studies, duration should therefore be read as a marker of cumulative clinical burden rather than as an independent driver of disability.

Finally, the structure of active comorbidities itself functions as a cross-cutting modifier. Whereas Section 3.2 identifies coexisting disorders as drivers to be recognized and managed in parallel with PPPD, the relevant operation here is different: it is the *configuration* of those comorbidities (which conditions remain simultaneously active, in what combination, and with what relative weight) that shapes how the phenotypic picture is expressed in the individual case (1). Empirical support comes from cohorts such as Waterston et al., in which patients meeting PPPD criteria displayed a heterogeneous distribution of coexisting conditions, including anxiety disorders, vestibular migraine, unilateral vestibular hypofunction, and isolated otolith abnormalities, often in combination rather than in isolation (4). Trinidade et al.’s systematic review further indicates that the relative weight of anxiety, autonomic arousal, body vigilance, and visual dependence often predicts persistent dizziness after peripheral vestibular insults more reliably than the severity of the original vestibular lesion (13). In practical terms, a comparable dominant exacerbating domain may express differently depending on whether an active migraine-related substrate, prominent affective load, or residual vestibular sensory abnormality is also operative, displacing the emphasis toward migraine-overlap, affective-hypervigilant, or mixed/multisensory features (4, 10). As with the other modifiers, this evidence is largely observational and should be read as a configuration that shifts clinical emphasis rather than as a determinant of categorical phenotype assignment. Thus, age, precipitant context, chronicity, and comorbidity structure refine the phenotypic picture; they do not replace it (1, 13).

### 4.4 Rationale for anchor phenotypes in clinical practice

From the standpoint of practical management, the key question is not whether PPPD already has a fully validated internal taxonomy, but whether clinicians can identify anchor phenotypes that make formulation and management more explicit (2). Such anchor phenotypes function as pragmatic reference points within a dimensional clinical space, making dominant symptom-generating and symptom-maintaining processes more visible in routine clinical practice (2). In conceptual terms, the three dimensional axes introduced in Section 4.2 (Three practical axes of phenotype-oriented formulation) and the anchor phenotypes developed in Section 5 (Anchor clinical phenotypes in PPPD) are complementary rather than competing descriptions: the axes map the continuous clinical space in which patients vary (by dominant exacerbating domain, degree of visual dependence, and affective-hypervigilant load) while the anchor phenotypes mark clinically recognizable patterns within that space and at its boundaries, each of which may be approached from a slightly different therapeutic angle.

It should be acknowledged at this point that the five anchor phenotypes proposed below are not mutually exclusive categories and are not all of the same ontological status, and the framework is more transparent when this heterogeneity is made explicit. Three of them (visually dominant, active motion–postural, and affective-hypervigilant) correspond to clinically recognizable regions of the dimensional space, each loosely aligned with predominance along one of the three axes. The mixed/multisensory phenotype, by contrast, is not a region of that space but a pattern of low differentiation across all axes, and its clinical identity rests on the absence of a single dominant provoking domain rather than on a distinct positive configuration. Finally, the migraine-overlap / VM–PPPD interface phenotype is not derived from the axes at all; it captures a pattern shaped by an active comorbid condition (vestibular migraine) that may modify any of the other configurations and therefore cuts transversally across the dimensional space. Acknowledging these three ontological roles (axis-aligned configuration, low-differentiation pattern, and transversal interface) clarifies what each anchor phenotype is doing in the framework and reinforces, rather than weakens, its clinical usefulness.

This strategy offers several practical advantages. First, it remains faithful to the current literature by preserving dimensional complexity while still allowing clinically recognizable patterns to emerge (1, 5). Second, it is communicatively useful: many patients understand their disorder more clearly when the clinician can explain not only that they have PPPD, but also which pattern appears to dominate their presentation (2, 3). Third, it provides a bridge to management, because each anchor phenotype may imply a different weighting of vestibular rehabilitation, psychoeducation, psychological integration, or migraine-oriented reasoning (1, 3, 5, 26).

For these reasons, the following section proposes five anchor clinical phenotypes in PPPD, organized around the three dimensional axes outlined above and further nuanced by cross-cutting modifiers, especially age and chronicity, while explicitly accommodating the residual and interface patterns described above. Their value lies in making PPPD more clinically actionable, not in implying that its internal structure has already been definitively resolved (1, 2, 3).

## 5 Anchor clinical phenotypes in PPPD

As developed in Section 4.4, the five anchor clinical phenotypes proposed here are not mutually exclusive categories and are not of uniform ontological status: visually dominant, active motion–postural, and affective-hypervigilant are axis-aligned configurations; mixed/multisensory is a low-differentiation pattern across all three axes; and the migraine-overlap / VM–PPPD interface is a transversal pattern shaped by active vestibular migraine comorbidity (10). Table 1 summarizes their defining clinical patterns and practical management implications.

**Table 1:** Anchor clinical phenotypes in PPPD and their practical management implications.

Anchor phenotype	Defining clinical pattern	Practical implications
Visually dominant/visually dependent	Visually complex or busy environments and dynamic visual settings dominate symptoms and disability.	Visually informed VRT and graded visual-motion exposure often merit early emphasis.
Active motion–postural	Self-motion, gait, turning, and upright tolerance dominate disability; residual vestibular sensory factors may contribute to symptom expression in some cases.	Motion-focused retraining and upright control may deserve greater emphasis; residual findings should not be overmedicalized.
Mixed/multisensory	Visual, motion, and postural provocations contribute relatively evenly to disability, without a single dominant exacerbating domain.	A hierarchized multimodal sequence is often more useful than a single-channel pathway.
Affective-anxious/hypervigilant	Anticipatory threat, body vigilance, avoidance, and maladaptive illness beliefs function as dominant maintenance mechanisms that amplify and perpetuate disability.	Early psychoeducation, reduction of safety behaviors, psychologically informed rehabilitation, CBT, and selected pharmacologic treatment may be particularly relevant.
Migraine-overlap/VM–PPPD interface	Persistent PPPD coexists with an active migraine background, VM as a precipitating event, or persistent interictal migrainous symptom modulation.	A PPPD-oriented rehabilitative frame should usually be maintained while active migraine drivers are addressed when clinically relevant.

Phenotypes are heuristic anchors of three ontological types within or adjacent to a continuous dimensional space defined by dominant exacerbating domain, visual dependence, and affective-hypervigilant load: visually dominant, active motion–postural, and affective-hypervigilant are axis-aligned configurations; mixed/multisensory is a low-differentiation pattern defined by the absence of a single dominant provoking domain; and migraine-overlap / VM–PPPD interface is a transversal pattern shaped by an active migraine-related comorbidity. Overlap is expected and dominant patterns may shift over time. The practical implications listed for each phenotype reflect clinically reasoned weighting of multimodal management rather than empirically validated phenotype-by-treatment interactions.

CBT, cognitive-behavioral therapy; PPPD, persistent postural-perceptual dizziness; VM, vestibular migraine; VRT, vestibular rehabilitation therapy.

### 5.1 Visually dominant/visually dependent phenotype

The visually dominant or visually dependent phenotype is one of the most clinically recognizable anchor phenotypes in PPPD. Its hallmark is disproportionate symptom aggravation in visually busy or visually dynamic environments, such as supermarkets, shopping malls, traffic, patterned floors, escalators, or screens. Patients often describe not only dizziness or non-spinning vertigo, but also perceptual overload, sensory disorganization, and difficulty tolerating visually complex settings that others navigate with little apparent effort (2, 26).

This phenotype is supported by converging, though still incomplete, evidence. Yagi et al. identified a visual-dominant cluster and found that the visual factor accounted for the largest proportion of variance in the Niigata questionnaire (5). In parallel, a nationwide Korean multicenter study comparing definite PPPD with clinical variant presentations highlighted the clinical relevance of visual exacerbation and suggested that visually weighted presentations may represent a meaningful clinical pattern within the PPPD spectrum (33). Chang and Schubert further showed that patients with PPPD reported greater visual vertigo than patients with VM across ecologically relevant real-world contexts (26). Emerging perceptual work adds a further nuance: compared with healthy controls, patients with PPPD required a higher proportion of coherent visual motion to determine motion direction, and this threshold increased with depressive burden (27). In addition, visual stress responses to static images that deviate from natural visual statistics have been associated with PPPD symptoms, suggesting that visually dominant presentations may involve broader atypical visual processing rather than only intolerance to overt visual motion (34). Taken together, these findings support the view that visual dependence may become a central organizing feature of the disorder in a subset of patients (5, 26, 27, 33, 34).

The clinical importance of this phenotype lies in its explanatory clarity. These patients may appear inconsistent because they function relatively well in simple environments yet deteriorate sharply under visual load. A phenotype-oriented formulation helps prevent that misreading and has an immediate practical implication: visually informed vestibular rehabilitation, graded exposure to visual motion, and explicit counseling about maladaptive visual weighting often merit particular emphasis in this subgroup of presentations (26). The point is not that these patients represent a biologically separate disease, but that their disability is organized around a clearly dominant perceptual domain.

### 5.2 Active motion–postural phenotype

The active motion–postural phenotype is centered on self-motion, gait, turning, head and body movement, and the maintenance of upright postural control. In these patients, bodily movement itself becomes effortful, destabilizing, or excessively salient, whereas visual provocation may still be present but is not the dominant complaint. This is the anchor phenotype that most closely corresponds to the active motion-dominant cluster described by Yagi et al. (5).

A related clinical nuance concerns residual vestibular sensory factors. Murofushi et al. reported frequent otolith dysfunction, including isolated otolith dysfunction, in patients meeting PPPD criteria, suggesting that otolith-related abnormalities may still be relevant to symptom expression in some motion–postural presentations (22). This interpretation is compatible with Yagi et al.’s observation that the active motion-dominant cluster was older than the visual-dominant cluster, a demographic pattern that may increase the likelihood of residual vestibular sensory contributions in this phenotype (5). These observations do not justify a separate otolithic subtype of PPPD, but they do support a more attentive clinical stance toward residual vestibular sensory abnormalities in motion–postural presentations.

From a practical perspective, this phenotype directs attention toward gait behavior, turning, upright tolerance, motion exposure, and the possibility that residual vestibular sensory factors may still modulate the disorder without fully accounting for it. It is particularly useful in patients whose PPPD followed an identifiable vestibular insult and whose current disability remains more strongly linked to self-motion than to visual scenes alone (1, 4, 22). In such cases, the phenotype-oriented formulation does not reduce PPPD to a residual lesion disorder, but it does help the clinician retain closer attention to the ways in which persistent functional symptoms and residual vestibular factors may still interact.

### 5.3 Mixed/multisensory phenotype

The mixed or multisensory phenotype refers to patients in whom no single exacerbating domain clearly dominates. Instead, visual motion, self-motion, upright posture, and broader perceptual challenge all contribute meaningfully and relatively evenly to the disorder. In Yagi’s cluster analysis, this was not merely a residual category but a recognizable pattern in its own right, although it is worth noting that it emerged from the merger of two initial clusters that differed in symptom severity but not in their exacerbation factor profile, reinforcing the interpretation that the mixed phenotype is defined by the absence of a single dominant provoking domain rather than by a distinct positive pattern (5).

This phenotype is important because it prevents over-simplification. Not all patients fit neatly into a visually dominant or motion–postural frame. For some, the most accurate clinical reading is that of a broader maladaptation of perceptual-postural control, in which several provoking domains have become similarly amplified. These patients often report being symptomatic across almost all provoking situations rather than identifying a single dominant trigger, and their disability may feel cumulative rather than tied to one specific context (2, 5).

The mixed phenotype is best understood as showing generalized amplification across multiple shared domains. That recognition is clinically useful because it points toward broader therapeutic prioritization and sequencing rather than a narrowly targeted first-line strategy (1, 5). In this sense, the phenotype should be viewed not as a diagnostic leftover, but as a legitimate and clinically informative pattern of evenly distributed multisensory burden.

### 5.4 Affective-anxious/hypervigilant phenotype

The affective-anxious/hypervigilant phenotype, also referred to in compact form as “affective-hypervigilant” throughout this review, is particularly relevant when symptom persistence, disability amplification, and treatment engagement are strongly shaped by anticipatory threat, body vigilance, avoidance, and maladaptive illness beliefs. In these patients, dizziness and fear of dizziness often become functionally inseparable (2). Importantly, this phenotype should not be equated with a formally diagnosable anxiety disorder, which may instead function as a precipitant, comorbidity, or active driver requiring treatment in its own right. The phenotype refers to a dominant configuration of cognitive-affective maintenance mechanisms that may be present with or without a concurrent anxiety diagnosis.

This formulation is supported by models of PPPD persistence that emphasize pre-existing anxiety vulnerability, anxiety-related responses to precipitating events, heightened body vigilance, and negative illness perceptions as plausible contributors to symptom persistence (3). Trinidade’s systematic review reached a closely aligned conclusion: following peripheral vestibular insults, anxiety, autonomic arousal, body vigilance, and visual dependence appear to be among the most plausible predictors of persistent dizziness, often more informative than the severity of the original vestibular lesion itself (13). More recent cross-sectional mediation analyses further suggest that psychological variables, particularly perceived injustice, anxiety, and balance vigilance, may shape the relationship between vestibular/perceptual symptoms and PPPD symptomatology (28). Large clinical series also support the practical relevance of this pattern, with anxiety disorders and related psychological comorbidities appearing prominently among the associated or precipitating conditions seen in routine PPPD cohorts (4).

Zhang et al. (29) add an important nuance by showing that emotional disorders were most frequent in middle-aged patients and less common in older adults, suggesting that affective loading may be unevenly distributed across PPPD presentations. Fukushima et al. (30) provide a complementary observation in an independent Japanese cohort, with younger patients showing higher anxiety scores on the Hospital Anxiety and Depression Scale than older patients. These observations should be interpreted cautiously, however, as they derive from retrospective single-center series in East Asian clinical populations, where cultural factors in emotional self-reporting may also contribute to the age-related difference observed. This does not mean that the affective-hypervigilant phenotype is confined to any single demographic group, but it does suggest that age and precipitant context may modulate how prominently it emerges (29, 30).

The practical importance of recognizing this phenotype is considerable. If the clinician focuses only on the original physical trigger, treatment may remain too narrow. By contrast, identifying the hypervigilant pattern reorients the therapeutic logic toward the cognitive-affective and behavioral mechanisms that plausibly sustain symptom persistence, with phenotype-specific management priorities developed in Section 8.4 (2, 3, 4). This phenotype should therefore be understood as a formulation of dominant maintenance mechanisms, not as a rebranding of PPPD into a psychiatric diagnosis.

### 5.5 Migraine-overlap/VM–PPPD interface phenotype

The migraine-overlap/VM–PPPD interface phenotype occupies the clinical borderland between PPPD and VM. It is most relevant in patients with a strong migraine background, VM as the precipitating event, persistent symptoms between attacks, or a mixture of visual sensitivity, motion intolerance, and migrainous features that complicate the clinical picture. As a clinical interface rather than a single disorder, it captures the situations in which the borderland between PPPD and VM becomes clinically central (10). The clinical plausibility of this interface is reinforced by case-series data showing VM among the most common associated or precipitating vestibular conditions in PPPD cohorts, supporting the view that this overlap is not exceptional but part of routine clinical reality (4).

Tarnutzer and Kaski (10) addressed this issue directly, arguing that when VM becomes persistent it is usually more coherent to classify the chronic disorder as PPPD, while emphasizing that VM and PPPD are not mutually exclusive and may lie along a broader episodic-to-chronic vestibular spectrum. This formulation is especially useful for phenotype-oriented reasoning because it helps avoid two symmetrical errors: reclassifying persistent PPPD too readily as “chronic VM,” or ignoring a genuine migrainous terrain that continues to shape symptom expression and treatment priorities (10).

Chang and Schubert (26) further support the relevance of this interface phenotype by showing that PPPD and VM differ in visual vertigo and motion-sickness profiles even though both may involve sensory hypersensitivity. Thus, overlap does not imply indistinguishability. Rather, it invites higher-resolution clinical formulation: is the persistent disorder more consistent with PPPD, more strongly influenced by migraine-related mechanisms, or shaped by both? (10, 26).

Phenotype-oriented formulation allows the VM–PPPD interface to be acknowledged without inflating it into a separate disease class (10). What matters clinically is not whether the interface can be purified into a single label, but whether migraine-related mechanisms remain sufficiently active to shape subsequent management.

## 6 Assessment tools for phenotype-oriented practice

### 6.1 Core clinical assessment

In PPPD, the core assessment remains fundamentally clinical (1, 3). The most informative first step is a directed history that establishes not only whether the patient fulfills positive diagnostic criteria, but also how symptoms are distributed across everyday situations and which provoking domains dominate the illness experience. This includes symptom chronology, precipitating event, daily persistence, functional impact, and the relative weighting of upright posture, active or passive motion, and visually complex environments as aggravators (1).

Within a phenotype-oriented framework, history-taking should also identify the degree of visual dependence, the presence of anticipatory threat, hypervigilance, avoidance and safety behaviors, relevant migraine features, and the burden of anxiety, depression, sleep disturbance, and reduced participation (3, 4, 35). This broader formulation matters because chronic disability in PPPD is often shaped not only by the provoking stimulus itself, but also by maladaptive perceptual weighting, body vigilance, negative illness beliefs, and symptom-focused behavior (2, 3, 4). For that reason, the clinical interview should move beyond the question of whether PPPD is present toward the more useful question of which symptom-generating and symptom-maintaining processes appear most dominant in that individual case (3).

Physical examination remains essential, but its role is best understood as focused and supportive rather than indefinitely exclusionary (1, 2). A reasoned vestibular and neurologic examination helps identify residual deficits, active comorbid drivers, and red flags requiring alternative investigation (1). At the same time, minor residual vestibular findings should not be allowed to eclipse the wider formulation when persistent disability appears to be driven mainly by visual dependence, mixed multisensory provocation, or affective-hypervigilant amplification (3, 4). In practice, the aim of assessment is not simply to confirm the disorder, but to generate a formulation that can guide subsequent management priorities.

### 6.2 Symptom and handicap measures in clinical follow-up

Questionnaires do not replace clinical synthesis, but they can make it more explicit, more reproducible, and more trackable over time (3). For global dizziness-related disability, the Dizziness Handicap Inventory (DHI) remains a pragmatic broad measure in routine follow-up because it captures functional, physical, and emotional burden in a format that is easy to repeat longitudinally (4, 12, 32). In PPPD populations, observational data also suggest that DHI is sensitive to the impact of chronicity, anxiety, and depression on perceived disability (12, 32), which makes it useful when the clinician is trying to distinguish symptom intensity from disability amplification. Table 2 summarizes the main questionnaire- and scale-based tools that can support phenotype-oriented clinical formulation and longitudinal follow-up.

**Table 2:** Questionnaire- and scale-based assessment tools supporting phenotype-oriented clinical formulation and longitudinal follow-up.

Assessment tool	What it adds	How it helps phenotype-oriented practice	Main caveat
DHI	Global physical, functional, and emotional dizziness-related handicap.	Tracks overall disability burden across follow-up; sensitive to the amplifying effects of chronicity, anxiety, and depression on perceived handicap.	Not phenotype-specific; does not profile dominant exacerbating domains.
NPQ	Relative burden across the three canonical exacerbating domains: upright posture/walking, movement, and visual stimulation.	Most useful for clarifying visual, movement, or mixed exacerbation weighting in phenotype-oriented profiling and longitudinal follow-up.	Supportive profiling and severity tool; standalone diagnostic accuracy is modest across Western and Asian validation studies.
ALQ	Symptom exacerbation across potential PPPD subtypes, including egomotion, rest, head motion, and visual motion contexts.	Emerging complement to the NPQ when explicit subtype profiling is desired; designed specifically to discriminate between potential phenotypes rather than quantify global severity.	Validation remains limited; best positioned currently as a research and emerging clinical tool.
HADS	Anxiety and depressive burden associated with symptom severity and treatment response.	Especially informative in affective-hypervigilant presentations; co-occurring high scores, anticipatory threat, and avoidance help objectify hypervigilant maintenance mechanisms.	Anxiety and depression may be present across all phenotypes and at varying severity; high scores alone do not define a phenotype.
HIT-6/MIDAS	Headache-related impact and migraine-related disability.	Useful in migraine-overlap/VM–PPPD interface presentations to quantify the active migraine component and to determine whether migraine-oriented management should be layered onto PPPD-oriented rehabilitation. Headache diaries may provide complementary longitudinal information on attack frequency, migrainous features, and acute medication use.	These tools do not assess PPPD severity or establish the diagnosis of PPPD; they should be interpreted only as measures of the migraine component of the formulation.
Complementary measures (ABC, WHOQOL-BREF, VVAS)	Balance confidence and fear of falling (ABC); broader health-related QoL and psychosocial burden (WHOQOL-BREF); visually induced dizziness profile (VVAS).	Selected according to dominant burden: ABC when balance confidence or fear of falling is prominent; WHOQOL-BREF when broader participation loss matters; VVAS when visual dependence profiling is needed.	Adjunctive tools; not recommended for routine use across all presentations.

All instruments listed are supportive clinical tools for formulation and follow-up. Their selection should be guided by the dominant burden and clinical question in each case. Repeated measurement supports detection of shifts in therapeutic priorities over time.

ABC, Activities-specific Balance Confidence scale; ALQ, Athens–Lübeck Questionnaire; DHI, Dizziness Handicap Inventory; HADS, Hospital Anxiety and Depression Scale; HIT-6, Headache Impact Test-6; MIDAS, Migraine Disability Assessment; NPQ, Niigata PPPD Questionnaire; PPPD, persistent postural-perceptual dizziness; QoL, quality of life; VM, vestibular migraine; VVAS, Visual Vertigo Analog Scale; WHOQOL-BREF, World Health Organization Quality of Life - Brief.

The Niigata PPPD Questionnaire (NPQ) offers a different and more phenotype-relevant type of information. Developed around the three canonical exacerbating domains of PPPD—upright posture or walking, movement, and visual stimulation (21)—it is particularly useful for clarifying the relative predominance of provoking factors in visually weighted, active motion–postural, or mixed presentations (5, 21). The NPQ is best regarded as a supportive profiling and follow-up measure rather than a standalone diagnostic instrument: its original development study showed good reliability and discrimination between PPPD and other vestibular disorders, with the visual stimulation factor having the greatest discriminatory value (21), but subsequent Spanish, French, and Korean validations have reported only modest standalone diagnostic accuracy, with the Korean study showing improved performance when NPQ dimensions were combined with DHI domains, particularly visual stimulation and emotional handicap (36, 37, 38). The revised German version (NPQ-R) supports clinical usefulness for severity assessment, although further refinement of its factor structure may still be warranted (39). Complementing these severity-oriented tools, the Athens–Lübeck Questionnaire (ALQ) has recently been introduced specifically to discriminate between potential PPPD subtypes, positioning it as an emerging complement to the NPQ for phenotype-oriented profiling rather than as a severity measure (40). Unlike the DHI, HADS, and NPQ, however, the ALQ has more limited validation to date and is at present best regarded as a research and emerging clinical instrument rather than as one ready for routine clinical use.

In selected patients, measures focused more specifically on visual dependence may add complementary value. In chronic dizziness cohorts, elevated Visual Vertigo Analog Scale scores characterized PPPD better than other balance or visual-dependence tests, although discriminative accuracy remained only fair; accordingly, such tools are best viewed as aids for profiling visually aggravated presentations and tailoring visual desensitization rather than as diagnostic tests in isolation (41).

Because affective burden frequently modulates severity and treatment response in PPPD, the Hospital Anxiety and Depression Scale (HADS) is also clinically useful as a structured follow-up measure (4, 12, 32). Higher anxiety and depression scores are associated with greater dizziness-related handicap and poorer quality-of-life outcomes (12, 32), which makes HADS especially relevant in patients with affective-hypervigilant presentations. In selected cases, complementary measures such as the Activities-specific Balance Confidence (ABC) scale or broader health-related quality-of-life instruments such as the World Health Organization Quality of Life - Brief (WHOQOL-BREF) may add further value, particularly when fear of falling, reduced participation, or broader psychosocial burden are prominent concerns (12, 35, 42).

When migraine overlap is clinically relevant, dizziness-specific and affective scales should be supplemented with measures of headache burden. The Headache Impact Test-6 (HIT-6) and the Migraine Disability Assessment (MIDAS) can document headache-related impact and disability, whereas headache diaries add longitudinal information on attack frequency, associated migrainous features, and acute medication use. These instruments do not assess PPPD severity and should not be used to support the diagnosis. In the VM–PPPD interface, their value is to clarify the migraine component of the formulation and whether migraine-oriented treatment should be added to PPPD-oriented rehabilitation.

### 6.3 Using assessment tools to support phenotype formulation

The value of these instruments becomes greatest when they are not interpreted in isolation, but reintegrated into the phenotype-oriented formulation outlined earlier. A profile with disproportionate NPQ visual stimulation burden, together with a history dominated by supermarkets, traffic, patterned floors, escalators, or screens, supports a visually dominant reading of the case (5, 21). A pattern in which gait, turning, head motion, and upright tolerance are more prominent than visual scenes may instead support an active motion–postural emphasis (5). Likewise, high HADS scores do not define an affective-hypervigilant phenotype on their own, but when they co-occur with marked anticipatory threat, self-monitoring, and avoidance, they help objectify the clinical weight of hypervigilant maintenance mechanisms (3, 4, 12).

Longitudinally, these tools are also useful because the dominant burden in PPPD may shift over time even when the syndromic diagnosis remains stable (3, 4). Over time, the dominant burden may shift from broad dizziness handicap toward more circumscribed visual intolerance, or from sensorimotor provocation toward persistent fear, reduced confidence, and avoidance (4, 32). Repeated DHI, NPQ, and HADS measurements therefore help the clinician not only quantify burden, but also detect when therapeutic priorities may need to be reweighted across follow-up (12, 32). Their role is best understood as supportive and structuring: they help objectify dominant patterns and monitor change, while the overall formulation remains grounded in expert history-taking and clinical examination (3, 5).

## 7 Multimodal management framework

### 7.1 Therapeutic communication and general principles

The first therapeutic intervention in PPPD is often the way the diagnosis is explained. Communication should frame PPPD as a real, recognizable, and potentially modifiable functional vestibular disorder, not as an unexplained symptom state or a diagnosis of exclusion (1, 2). Persistent fears about missed structural disease, falling, or irreversible deterioration may themselves become active barriers to recovery (2, 43). At the same time, reducing PPPD to “stress” or to something “all in the mind” is equally unhelpful, since it ignores the disorder’s sensorimotor and perceptual features and often alienates patients whose symptoms are involuntary and disabling (2, 4).

A constructive explanation links symptoms to maladaptive but potentially reversible changes in sensory weighting, postural control, visual dependence, and threat-related self-monitoring, framed as a self-perpetuating but non-threatening process rather than as signs of ongoing structural harm (3, 4, 43). Concrete perceptual analogies can make this explanation more intuitive: the familiar illusion of self-motion felt when a neighboring train starts to move while one’s own train is stationary illustrates how the brain can misattribute visual motion to the self, and how sensory reweighting, rather than structural damage, can generate compelling yet non-threatening symptoms. Such analogies can demystify the disorder and reduce the anticipatory threat that sustains it (43). These constructs are currently proposed maintenance mechanisms rather than elements of the Bárány Society criteria (see Section 3.1), and they create a rationale for treatment: if the disorder is maintained by maladaptive patterns of perception, behavior, and sensorimotor control, then rehabilitation, graded exposure, behavioral change, and multimodal support become more intelligible and credible (1, 2, 44).

Effective communication also means aligning expectations. Improvement in PPPD is typically gradual and cumulative rather than immediate, and the aim is restoration of function and reduced disability rather than the abrupt disappearance of all bodily sensations (3, 11).

Current PPPD literature supports a multimodal clinical approach reflecting the heterogeneity of the underlying evidence base (11, 15, 16, 44). This is conceptually consistent with models emphasizing interacting sensory, postural, visual, affective, and behavioral mechanisms rather than one isolated driver alone. It is also consistent with clinical series and interdisciplinary treatment reports in which benefit is typically sought through combinations of vestibular rehabilitation, psychoeducation or cognitive-behavioral intervention, and, in selected cases, pharmacologic treatment rather than through a single modality in isolation (4, 44). Accordingly, the role of phenotyping in PPPD is not to create separate treatment pathways, but to help reweight, sequence, and integrate available modalities. Different presentations may bring particular components to the foreground, but none of them abolishes the need for a multimodal plan.

Across phenotypes, several treatment goals remain constant. The first is reduction of disability rather than reduction of symptom salience alone. PPPD becomes clinically important because it restricts behavior, mobility, participation, confidence, everyday functioning, and quality of life (12, 35). The second is restoration of tolerance to the key provoking domains of the disorder: upright posture, self-motion, and visually complex environments (1, 21). The third is reduction of maladaptive avoidance, threat monitoring, and safety behaviors, particularly in patients whose illness course has become dominated by fearful expectation and self-observation (3, 4). A fourth goal is the active treatment of relevant comorbid drivers. These may include VM, residual vestibular hypofunction, panic symptoms, depressive burden, sleep disturbance, or fear of falling (4, 10, 12). Framing such factors as active drivers rather than as passive background comorbidities is useful because it emphasizes that they may shape outcome if left untreated. Thus, even before phenotype-specific prioritization begins, multimodal management in PPPD should aim to restore function, reduce amplification, and address whichever perpetuating mechanisms remain clinically relevant.

### 7.2 Vestibular rehabilitation as a central sensorimotor intervention

Vestibular rehabilitation therapy (VRT) remains the most consistently central modality across PPPD phenotypes, although its role is better understood in broader terms than classic peripheral vestibular compensation alone. In PPPD, VRT often functions as a process of sensorimotor recalibration, graded exposure, and restoration of tolerance to motion, posture, and visually complex environments (2, 3). This interpretation is compatible with current models of PPPD as a chronic functional vestibular disorder involving maladaptive interactions among vestibular, visual, postural, attentional, and affective processes (3).

From a phenotype-oriented perspective, what changes is not whether VRT is relevant, but what it is mainly trying to accomplish, for example, graded visual desensitization in visually dominant patients versus retraining of gait, turning, and self-motion tolerance in active motion–postural presentations. VRT is therefore transversal across phenotypes, while its therapeutic emphasis is not identical across them (43, 44); the phenotype-specific applications are detailed in Section 8 (Phenotype-oriented practical management).

The literature also suggests that the delivery format of VRT may vary without changing its conceptual core, but that the conceptual framing matters. A prospective comparison from the Niigata group directly addressed this issue by applying the same structured vestibular rehabilitation program to patients with chronic unilateral vestibular hypofunction and to patients with PPPD treated at the same center. In the hypofunction group, the program produced significant reductions in dizziness handicap, in most Niigata PPPD Questionnaire (NPQ) domains, and in anxiety. In the PPPD group, by contrast, the same program produced no significant reductions in the DHI total or the HADS, achieved reductions in only one of the three NPQ subscales (motion), and was associated with a substantially higher rate of early discontinuation owing to symptom exacerbation (45). Although sample size was limited, this observation reinforces the view that VRT in PPPD cannot be applied as a direct transposition of peripheral compensation protocols and supports the need for a phenotype-oriented therapeutic rationale.

Consistent with this view, recent prospective, pilot, randomized, and meta-analytic data nevertheless support clinically meaningful short-term improvement with VRT in PPPD, including reductions in dizziness-related handicap and, in some studies, anxiety burden. These benefits are observed across structured clinic-based, home-based, customized, and virtual-reality- and optokinetic-assisted formats. Randomized comparisons, however, suggest that adding virtual reality to a structured VRT-plus-optokinetic program does not necessarily confer clear between-group superiority (20, 46, 47, 48, 49). Home-based programs, clinic-based regimens, optokinetic augmentation, and virtual-reality-assisted exposure therefore converge on the principle of repeated, tolerable exposure that promotes recalibration rather than withdrawal (12, 50). At present, however, the evidence remains heterogeneous, and no specific format can be regarded as clearly superior across all PPPD presentations (15, 18). For that reason, within a phenotype-oriented framework, VRT is best regarded as a central sensorimotor component of treatment, while its exact content and emphasis remain adaptable to the dominant clinical pattern.

### 7.3 Cognitive-behavioral and psychologically informed interventions

If VRT represents the principal sensorimotor modality in PPPD, cognitive-behavioral and psychologically informed interventions are the main means of addressing the interpretive, attentional, and behavioral mechanisms that often sustain chronic symptoms. Across the literature, these interventions include psychoeducation, formal cognitive-behavioral therapy (CBT), graded exposure, work on safety behaviors and avoidance, and, increasingly, forms of vestibular rehabilitation explicitly informed by cognitive-behavioral principles (2, 43, 44).

Their relative role, however, is not uniform across phenotypes; for example, psychologically informed care may take a leading role in affective-hypervigilant presentations, whereas in visually dominant or active motion–postural cases it more often functions as facilitator of exposure, engagement, and adherence. As with VRT, the modality itself is not phenotype-specific, but its clinical weight and timing may change meaningfully across patterns of presentation (43, 44); the phenotype-specific applications are detailed in Section 8 (Phenotype-oriented practical management).

A further transversal point is that CBT-informed care and VRT are often best viewed as interacting rather than parallel modalities. Several practical and review-level sources suggest that vestibular rehabilitation may be more effective when the patient’s threat interpretation, avoidance, and behavioral responses are addressed at the same time as motion and postural tolerance (2, 17, 44). This general principle is reinforced by Waterston’s clinical series, in which many patients were managed with combined CBT, antidepressant treatment, and continuation of vestibular exercises rather than with a single isolated modality, and in which CBT was associated with substantial improvement in dizziness, handicap, and anxiety measures over follow-up (4). This observation supports phenotype-oriented weighting of care without implying that the patient is being moved from a “vestibular” pathway into a wholly separate “psychological” one (4). Rather, it suggests that the relationship between these modalities is often complementary and dynamic.

### 7.4 Pharmacologic treatment as supportive, phenotype-oriented care

Pharmacologic treatment occupies a more supportive and selective place in PPPD than vestibular rehabilitation or psychoeducational/behavioral care. Current literature continues to regard SSRIs and SNRIs as plausible adjuncts, especially when affective burden, hypervigilance, sleep disturbance, or migraine-overlap features are prominent, but the evidence base remains limited and does not justify strong phenotype-stratified recommendations (3, 14). Older open-label studies and subsequent observational series suggest that serotonergic medication may improve dizziness-related handicap in some patients, but these data derive largely from uncontrolled or combined-treatment designs and do not justify strong phenotype-stratified recommendations under modern PPPD criteria (4, 51). Consistent with the broader evidence picture introduced earlier, the 2023 Cochrane review identified no placebo-controlled randomized trials of SSRIs or SNRIs meeting Bárány Society criteria, which constrains the strength of any phenotype-specific pharmacologic recommendation in current practice (14).

Within a phenotype-oriented framework, pharmacotherapy is therefore best interpreted as an adjunctive treatment option whose plausibility increases when specific maintaining factors are prominent. In affective-hypervigilant cases, serotonergic medication may be easier to justify because anxiety, vigilance, and persistent threat expectation contribute materially to disability. In migraine-overlap cases, pharmacologic treatment may also be relevant when active migraine-related mechanisms remain clinically important. In more clearly visual or motion-dominant presentations, medication is often better understood as a facilitator of rehabilitation than as the leading therapeutic logic (10, 43).

This more modest positioning is methodologically preferable. It allows acknowledgement of the longstanding clinical use of serotonergic medication in chronic dizziness and PPPD-related states while avoiding any suggestion that pharmacologic treatment has already been validated as a phenotype-specific intervention. In the context of the present review, medication is therefore best understood as supportive, phenotype-informed care rather than as a formal treatment stratum.

### 7.5 Practical sequencing, escalation, and follow-up

Across phenotypes, a reasonable practical sequence begins with clear diagnostic communication and early vestibular rehabilitation, followed (when needed) by tighter integration of psychologically informed care and selective pharmacologic treatment (2, 43).

From a phenotype-oriented perspective, escalation should not be driven only by lack of response, but also by reformulation of what now dominates the disorder. The logic of escalation is therefore not simply intensification, but reweighting (10, 43).

Follow-up should remain longitudinal and formulation-sensitive, because improvement in PPPD is often nonlinear and partial gains in one domain may uncover other active drivers. Repeated review should therefore reassess whether the therapeutic balance of the plan needs to shift (4, 12). Taken together, the practical logic of phenotype-oriented management is not one of rigid algorithms but of iterative clinical reasoning: diagnosis establishes the disorder, formulation identifies the dominant drivers, and repeated reassessment keeps the therapeutic plan aligned with the evolving clinical picture.

**Figure 1:**
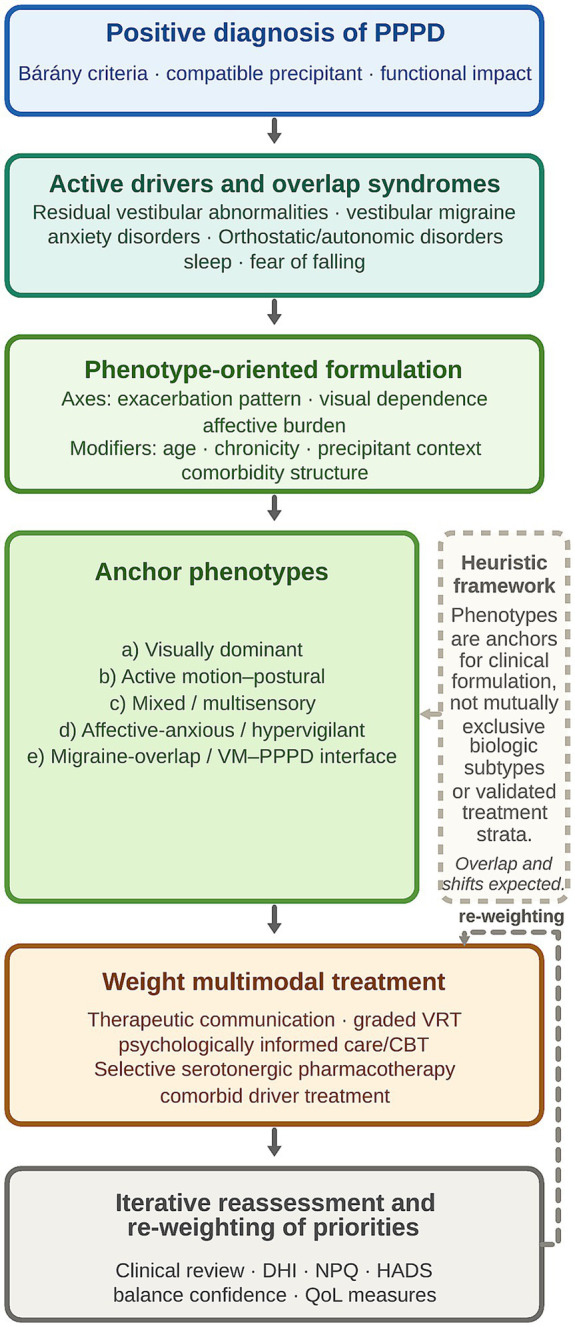
Practical framework map for phenotype-oriented management in persistent postural-perceptual dizziness (PPPD). The figure summarizes the main components of the clinical reasoning process proposed in this review. A positive diagnosis according to the Bárány Society criteria provides the entry point for care. The clinician then identifies active comorbid drivers and overlap syndromes, formulates the case using dominant clinical dimensions and cross-cutting modifiers, and recognizes one or more anchor phenotypes that may guide the weighting of multimodal treatment. The arrows indicate a common direction of clinical reasoning rather than an obligatory linear sequence, and the dashed loop emphasizes that reassessment may lead to iterative re-weighting of priorities over time. Anxiety disorders in the “active drivers and overlap syndromes” component refer to formally diagnosable comorbid entities requiring specific treatment and are conceptually distinct from the affective-hypervigilant phenotype, which describes a dominant configuration of cognitive-affective maintenance mechanisms and may be present with or without a formal anxiety diagnosis. The framework is presented as a pragmatic clinical heuristic, not as a validated treatment-stratification algorithm. CBT, cognitive-behavioral therapy; DHI, Dizziness Handicap Inventory; HADS, Hospital Anxiety and Depression Scale; NPQ, Niigata PPPD Questionnaire; PPPD, persistent postural-perceptual dizziness; QoL, quality of life; VM, vestibular migraine; VRT, vestibular rehabilitation therapy.

Figure 1 summarizes this framework map, linking positive diagnosis, identification of active comorbid drivers, phenotype-oriented formulation within a dimensional framework, weighting of multimodal care, and iterative reassessment.

## 8 Phenotype-oriented practical management

### 8.1 Management priorities in the visually dominant / visually dependent phenotype

Within the multimodal framework outlined in Section 7 (*Multimodal management framework*), management of the visually dominant phenotype is distinguished by two practical emphases.

The first is a graded hierarchy of visual exposure, beginning with lower-intensity stimuli or shorter durations and progressing toward richer and more immersive contexts. Depending on local resources, this may involve controlled video-based motion, optokinetic paradigms, structured walking in increasingly busy environments, or virtual-reality-assisted exposure (46, 50). Naturalistic settings that combine an uneven support surface with peripheral optic flow, such as walking on natural outdoor terrain, can offer an accessible, low-technology form of the same combined somatosensory and optokinetic challenge, conceptually analogous to the exposure sought in busy visual environments such as shopping malls. Excessive early provocation in this phenotype is particularly likely to reinforce fear and avoidance rather than recalibration, so progression should be patient-centered. Because patients with PPPD differ from those with VM in the intensity and ecological profile of visual vertigo, this emphasis on explicit visual desensitization is more coherent than assuming that all visual sensitivity in chronic dizziness is fundamentally migrainous (26).

The second is explicit work on visually patterned avoidance and protective behaviors. Many patients narrow their environments, over-monitor visual triggers, or adopt compensatory habits that paradoxically sustain visual dependence. Visually oriented rehabilitation is therefore likely to be more effective when paired with coaching about tolerable symptom activation, abandonment of unnecessary safety behaviors, and graded re-entry into relevant environments (2, 3). When anticipatory anxiety or marked avoidance is also prominent, earlier integration of psychologically informed care (Section 7.3, *Cognitive-behavioral and psychologically informed interventions*) is justified, not because the phenotype ceases to be visual, but because visual exposure is much less effective when threat processing dominates the response to it (2).

### 8.2 Management priorities in the active motion–postural phenotype

In the active motion–postural phenotype, the same multimodal toolkit is reweighted toward self-motion, gait, turning, and upright control rather than visual desensitization. The rehabilitative target is not balance in the abstract but the excessive salience and perceived threat of ordinary movement: some patients adopt stiffened or overcontrolled movement strategies that preserve short-term stability at the cost of long-term maladaptation, and helping them relinquish these rigid, fear-linked patterns is a phenotype-specific objective (3, 52). Recent prospective observational data support short-term improvements in physical, functional, and emotional handicap with this orientation, sustained at 3 months (48).

This phenotype also warrants explicit consideration of residual vestibular sensory factors, particularly when PPPD followed a clearly identifiable vestibular insult. However, in the management phase, such findings should be used to tailor motion and posture retraining rather than to reclassify the disorder as a residual lesion syndrome.

The methodological caution that follows is to avoid overmedicalizing residual findings. Subtle vestibular abnormalities do not abolish the diagnosis of PPPD nor remove the need for habituation-based and psychologically informed management; the most useful formulation is usually that a residual vestibular driver still contributes to symptom expression, but the persistent disorder now requires active motion and posture retraining within a functional vestibular framework (1, 22). Where anxiety is clearly secondary rather than dominant, pharmacologic escalation is less relevant than structured physical retraining.

### 8.3 Management priorities in the mixed/multisensory phenotype

The mixed/multisensory phenotype is distinctive not for foregrounding one channel of treatment but for requiring disciplined prioritization across several. Rather than forcing the patient into an artificially pure visual or motion-based pathway, the clinician identifies which combinations of triggers most strongly shape everyday disability and sequences treatment accordingly (2, 5). Modular sequencing usually serves better than diffuse simultaneity: one patient may need visual desensitization first because shopping environments are the most disabling; another may need early emphasis on turning and gait because fear of self-motion dominates daily restriction. Without such sequencing, treatment can become excessively broad, fatiguing, and conceptually unclear to the patient, threatening adherence (12, 44).

Expectation management is also particularly important in this phenotype. Because several provoking domains are active at once, improvement may be slower and more nonlinear, and progress in one domain may initially unmask the burden of another. Follow-up is therefore most useful when it explicitly reassesses which component now dominates disability rather than assuming that the original treatment emphasis remains correct throughout care (12, 43).

### 8.4 Management priorities in the affective-anxious/hypervigilant phenotype

In the affective-hypervigilant phenotype, weighting shifts earlier toward psychologically informed care (Section 7.3) and toward a comparatively lower threshold for selective serotonergic pharmacotherapy (Section 7.4, *Pharmacologic treatment as supportive, phenotype-oriented care*). Movement, posture, and visual exposure remain necessary, but they are likely to be most effective when framed and paced in a way that actively targets fear, expectancy, and avoidance: the patient is not only practicing movement, but learning that symptom activation can be tolerated and reinterpreted without escalating threat responses (2, 3, 13). The quality of the diagnostic explanation matters disproportionately here, because hypervigilant patients’ willingness to engage with exposure-based treatment and to relinquish safety behaviors depends on it (43).

Selective serotonergic pharmacotherapy may also deserve earlier consideration in this phenotype, particularly when affective burden, hypervigilance, or persistent anticipatory threat are clinically prominent. The clinical plausibility of SSRIs/SNRIs is not restricted to patients with formal psychiatric diagnoses, and observational work suggests that serotonergic treatment may improve dizziness outcomes in some PPPD patients even when overt psychiatric symptoms are not the most obvious presenting feature, although these data remain observational, often combined-treatment, and not strong enough to support phenotype-specific prescribing rules (51, 53). What this phenotype offers is therefore not new evidence but a more easily justified prescribing context, given the salience of the affective-hypervigilant loop (3).

### 8.5 Management priorities in the migraine-overlap/VM–PPPD interface phenotype

In the migraine-overlap phenotype, the central practical task is to layer two therapeutic logics rather than choose between them. The first practical requirement is phenomenologic clarification: the clinician must distinguish what represents active episodic migraine activity, what is more consistent with persistent PPPD, and what role migraine-related sensory hypersensitivity continues to play in the chronic disorder. Treatment becomes confused when persistent PPPD is managed as though it were ongoing VM, or when substantial migraine-related mechanisms are ignored once the patient meets PPPD criteria (10, 54).

PPPD-oriented rehabilitation (Section 7.2, Vestibular rehabilitation as a central sensorimotor intervention) usually remains the backbone of management once the disorder fits PPPD; Moreno-Ajona’s review supports this distinction, with PPPD treatment centered on vestibular rehabilitation and multidisciplinary care, whereas VM responds to migraine-directed strategies (54). When the patient continues to experience clear migrainous attacks, migraine triggers, or symptom modulation compatible with active migraine-related mechanisms, preventive or symptomatic migraine therapy may nonetheless be appropriate, as management of an active migraine comorbidity, not as a phenotype-specific intervention for PPPD itself. Supporting evidence derives from vestibular migraine populations rather than from validated PPPD or PPPD–VM interface cohorts, and may include conventional preventive treatments such as flunarizine where available and emerging calcitonin gene-related peptide-targeted agents, although the role of these treatments in the overlap phenotype specifically remains to be established (10, 54).

Affective burden also frequently warrants attention, because anxiety is common in both PPPD and chronic migraine states. Interface cases may therefore require a three-layered strategy: PPPD-oriented rehabilitation (Section 7.2), migraine-oriented management when active migraine-related mechanisms are present, and psychologically informed care (Section 7.3) when vigilance, distress, or avoidance become significant chronifying factors. The challenge is not diagnostic purity, but adequate treatment of all relevant drivers (10).

### 8.6 Overlap cases and phenotype transitions in clinical practice

In real practice, many patients do not remain stably within one phenotype: overlap and shifts in dominant maintenance loops are expected, and are themselves influenced by age, chronicity, and precipitant context (12, 29, 43). Phenotype-oriented management is therefore best understood as a dynamic prioritization framework rather than a once-and-for-all assignment, with cross-cutting modifiers as part of the reason why ongoing reformulation remains necessary, not as a weakness of the framework.

## 9 Discussion

The conceptual contribution of this review lies in clarifying the operational role of anchor phenotypes within a dimensional formulation framework. The framework operates at the level of clinical formulation rather than diagnosis or biology: it does not redefine what PPPD is, but renders more transparent how the diagnosis can be translated into a prioritized management plan, while remaining fully consistent with Bárány Society criteria as the entry point of care. Its specific contribution is to make explicit a reasoning process that experienced clinicians already perform implicitly when they decide which exacerbating domain to address first, how strongly to weight visual desensitization or motion retraining, and when to integrate psychologically informed care or selective serotonergic pharmacotherapy.

This positioning differentiates the present review from existing syntheses without competing with them. Consensus and conceptual reviews have established what PPPD is and how it should be diagnosed (1, 2, 3); recent treatment-oriented syntheses, including the 2023 Cochrane reviews, have characterized the available pharmacological and non-pharmacological evidence base and its limits (11, 14, 15); commentary and overlap-focused work has clarified the relationship between PPPD and vestibular migraine (10, 54); and recent practical guidance has emphasized communication, formulation, and bedside reasoning (43). The contribution attempted here sits between these layers: it accepts the diagnostic construct, takes the heterogeneous evidence base as it is, and proposes a clinically reasoned way of weighting and sequencing available modalities according to dominant clinical pattern. In that sense, the framework is complementary to (rather than a substitute for) diagnostic, pathophysiologic, and treatment-evidence reviews of PPPD.

The practical implications for neuro-otology follow from this positioning. First, communication and formulation become explicit therapeutic tools rather than implicit professional habits: the way the diagnosis is delivered, and the way dominant exacerbating domains are named back to the patient, can themselves shape engagement with rehabilitation, willingness to relinquish safety behaviors, and tolerance of graded exposure. Second, treatment selection becomes less about choosing between modalities and more about calibrating their relative emphasis and timing according to the dominant clinical pattern, which gives clinicians a transparent rationale to share with patients and a more legible framework within which to escalate, de-escalate, or reweight care over time. Third, the framework supports a longitudinal stance in which reassessment is not only a check on improvement but also an opportunity to reformulate the dominant burden, recognizing that what most disables a patient at 3 months may not be what most disables them at twelve. None of these implications introduces new clinical actions; what changes is the explicitness with which existing actions are reasoned, prioritized, and communicated.

### 9.1 Future directions

A central implication of the present review is that phenotype-oriented reasoning is clinically useful, but not yet prospectively validated as a stratification framework. Current evidence supports recurring patterns of visual predominance, active motion–postural weighting, mixed multisensory amplification, affective-hypervigilant loading, and migraine overlap (3, 5, 10). However, these patterns remain heuristic unless future studies show that they predict clinically meaningful differences in clinical course, disability burden, functional outcome, treatment needs, or response profiles (13). The next step is therefore not simply to refine phenotype labels, but to determine whether such formulations have prospective prognostic, explanatory and therapeutic value.

This will require longitudinal rather than predominantly cross-sectional research. Patients should be phenotyped early using reproducible clinical and questionnaire-based criteria and then followed to determine whether dominant patterns remain stable, shift over time, or converge under treatment (5, 32). Such work would help answer several unresolved questions: do visually dominant cases show different functional outcomes from affective-hypervigilant ones; do active motion–postural cases more often retain residual vestibular abnormalities; and does the VM–PPPD interface differ from non-migraine presentations in course or treatment needs (10, 22).

Several research priorities follow directly from this framework. First, treatment studies should move beyond undifferentiated PPPD cohorts whenever feasible. Current interventional literature remains heterogeneous in modality, comparator choice, and outcome reporting (15, 16, 19). Stratified, or at least phenotype-sensitive, trials would help determine whether dominant clinical patterns interact meaningfully with treatment response. For example, visually dominant cases could reasonably motivate trials emphasizing visual-motion exposure and visually enriched vestibular rehabilitation, whereas affective-hypervigilant cases could motivate greater evaluation of psychologically informed rehabilitation, CBT-enriched approaches, or different timings of pharmacologic augmentation (16, 55).

Second, future studies should not focus only on whether a phenotype “matches” one intervention, but on how multimodal care can be better weighted and sequenced according to the dominant presentation. This is especially important because current practice rarely relies on a single modality in isolation (4, 44). Research should therefore examine whether phenotype-informed formulation improves the efficiency, timing, or sequencing of multimodal care, for example by identifying which patients may benefit from earlier behavioral integration, earlier visual desensitization, or more explicit treatment of residual migraine-related mechanisms.

Third, the PPPD–VM interface deserves dedicated longitudinal study, not only to clarify its clinical boundaries, but also to determine whether overlap cases differ in clinical course, treatment sequencing, or response to combined PPPD-oriented and migraine-oriented care (10, 26, 54). This line of research is particularly important because the interface is clinically meaningful even if it does not justify a separate disease category.

Fourth, future studies should give greater priority to outcomes that matter in practice. Symptom severity scales remain useful, but function, participation, balance confidence, quality of life, and durability of response may be more informative for practical management than symptom intensity alone (12, 35). This is especially relevant in a disorder in which disability is often amplified by avoidance, fear, and behavioral contraction. If future studies show that phenotype-informed formulation improves treatment selection, sequencing, or functional outcome in real practice, then the framework proposed here will have moved closer to a genuinely testable model of clinically useful stratification.

### 9.2 Limitations

The evidence base underlying phenotype-oriented reasoning in PPPD remains heterogeneous. It draws on consensus documents, narrative reviews, cross-sectional studies, exploratory clustering analyses, observational clinical series, and treatment studies with variable comparators, outcome measures, and follow-up durations (1, 3, 4, 5, 11, 13, 14, 15, 16, 18, 19). Direct evidence linking particular phenotypic configurations to differential prognosis or treatment response remains limited. Much of the available therapeutic literature evaluates mixed PPPD cohorts, multimodal interventions, or broader chronic dizziness populations, making interaction between dominant clinical pattern and treatment effect difficult to determine with confidence (16, 18, 19). Accordingly, some of the management implications discussed in this review remain inferential rather than being directly supported by phenotype-stratified trials.

The present framework also has limitations of its own. This is a clinically focused narrative review rather than a systematic review or formal guideline, and the synthesis was intentionally weighted toward clinical applicability rather than exhaustive evidence mapping. That choice improves practical usefulness, but also introduces an interpretive bias toward clinically recognizable patterns and management-relevant concepts. The proposed phenotypes should accordingly be understood as heuristic anchors for formulation and prioritization rather than as exhaustive, mutually exclusive, or prospectively validated treatment strata: boundaries between phenotypes are fluid, overlap is common, and dominant patterns may shift over time (3, 11).

Several of the empirical pillars that most directly inform the proposed framework come from studies with modest sample sizes, and this should be made explicit. The canonical exploratory clustering of exacerbation patterns derives from a single-center cohort of 108 untreated patients with PPPD, in which conventional vestibular tests, DHI scores, and HADS scores did not differ significantly between the identified subtypes, with age being the only demographic dimension that separated visual-dominant from active motion-dominant clusters (5). Controlled motion-perception data that refine the visual-dependence axis come from a cohort of 32 patients with PPPD and 28 age-matched controls (27), and the direct comparison between structured vestibular rehabilitation in chronic unilateral vestibular hypofunction and in PPPD that anchors part of the discussion on modality framing in Section 7.2 derives from a single-center prospective study of 19 patients with chronic unilateral vestibular hypofunction and 15 patients with PPPD, in which 6 of the 15 PPPD participants discontinued vestibular rehabilitation owing to symptom exacerbation, leaving treatment-effect estimates in PPPD based on a self-selected subgroup of tolerators (45). These sample-size and self-selection constraints reinforce the reading that current support for dimensional heterogeneity, subtype-like patterns, and modality-specific tolerability in PPPD is suggestive rather than definitive.

The present synthesis may also carry an interpretive bias toward data that are congruent with a phenotype-oriented reading of PPPD; some findings complicate that reading and deserve particular emphasis in future work, including the perceptual observation that patients with PPPD required greater, not lesser, motion coherence to identify direction of visual motion, a finding that qualifies any simple narrative of “visual over-reliance” (27). Clinical epidemiology also tempers the weight of precipitant context as an organizing variable, since large registry-based comparisons have suggested that primary and secondary chronic functional dizziness present with broadly similar symptom profiles and disability burdens (31).

Taken together, these caveats do not undermine the usefulness of anchor phenotypes as clinical heuristics, but they do frame the proposed framework as a starting point for prospective phenotype-sensitive research rather than as a closed empirical construct.

It should also be noted that, although prospective predictive evidence links anxiety-related mechanisms, body vigilance, and visual dependence to persistence of dizziness after peripheral vestibular insults (13), experimental evidence for true causal effects of these mechanisms on outcome remains limited, and the language used throughout this review to describe their role is deliberately framed in terms of contributing and predictive rather than determining factors.

## 10 Conclusion

PPPD can be reliably diagnosed using the Bárány Society criteria, but diagnosis is only the starting point of competent care. The decisive clinical step is formulation: making explicit which provoking domain dominates, which maintenance mechanisms sustain chronicity, and which comorbid drivers remain active in the individual patient. The phenotype-oriented framework proposed here operationalizes this step, indicating how vestibular rehabilitation, cognitive-behavioral and other psychologically informed interventions, migraine-oriented management, and selected pharmacologic treatment may be clinically weighted, sequenced, and reformulated as the dominant burden evolves. Its practical contribution is to translate the internal heterogeneity of PPPD into a transparent and individualized clinical plan, anchoring everyday neuro-otology practice in something more useful than the diagnostic label alone.

## References

[1] Staab JP Eckhardt-Henn A Horii A Jacob R Strupp M Brandt T . Diagnostic criteria for persistent postural-perceptual dizziness (PPPD): consensus document of the Committee for the Classification of vestibular disorders of the Bárány society. J Vestib Res. (2017) 27:191–208. doi: 10.3233/VES-170622, 29036855 PMC9249299

[2] Popkirov S Staab JP Stone J. Persistent postural-perceptual dizziness (PPPD): a common, characteristic and treatable cause of chronic dizziness. Pract Neurol. (2018) 18:5–13. doi: 10.1136/practneurol-2017-001809, 29208729

[3] Staab JP. Persistent postural-perceptual dizziness. Semin Neurol. (2020) 40:130–7. doi: 10.1055/s-0039-3402736, 31935771

[4] Waterston J Chen L Mahony K Gencarelli J Stuart G. Persistent postural-perceptual dizziness: precipitating conditions, co-morbidities and treatment with cognitive behavioral therapy. Front Neurol. (2021) 12:795516. doi: 10.3389/fneur.2021.795516, 35027907 PMC8749949

[5] Yagi C Morita Y Kitazawa M Yamagishi T Ohshima S Izumi S . Subtypes of persistent postural-perceptual dizziness. Front Neurol. (2021) 12:652366. doi: 10.3389/fneur.2021.652366, 33935950 PMC8085253

[6] Lee JO Lee ES Kim JS Lee YB Jeong Y Choi BS . Altered brain function in persistent postural perceptual dizziness: a study on resting state functional connectivity. Hum Brain Mapp. (2018) 39:3340–53. doi: 10.1002/hbm.24080, 29656497 PMC6866559

[7] Yagi C Morita Y Yamagishi T Ohshima S Izumi S Takahashi K . Changes in functional connectivity among vestibulo-visuo-somatosensory and spatial cognitive cortical areas in persistent postural-perceptual dizziness: resting-state fMRI studies before and after visual stimulation. Front Neurol. (2023) 14:1215004. doi: 10.3389/fneur.2023.1215004, 37554393 PMC10406134

[8] Li K Ling X Zhao J Wang Z Yang X. Abnormal neural circuits and altered brain network topological properties in patients with persistent postural-perceptual dizziness. Commun Biol. (2025) 8:122. doi: 10.1038/s42003-024-07375-z, 39863736 PMC11762978

[9] Indovina I Passamonti L Mucci V Chiarella G Lacquaniti F Staab JP. Brain correlates of persistent postural-perceptual dizziness: a review of neuroimaging studies. J Clin Med. (2021) 10:4274. doi: 10.3390/jcm10184274, 34575385 PMC8468644

[10] Tarnutzer AA Kaski D. What’s in a name? Chronic vestibular migraine or persistent postural perceptual dizziness? Brain Sci. (2023) 13:1692. doi: 10.3390/brainsci13121692, 38137140 PMC10741489

[11] Trinidade A Cabreira V Kaski D Goebel JA Staab JP Popkirov S . Treatment of persistent postural-perceptual dizziness (PPPD). Curr Treat Options Neurol. (2023) 25:281–306. doi: 10.1007/s11940-023-00761-8

[12] Alahmari KA Alshehri S. Evaluating the efficacy of vestibular rehabilitation therapy on quality of life in persistent postural-perceptual dizziness: the role of anxiety and depression in treatment outcomes. Front Neurol. (2025) 16:1524324. doi: 10.3389/fneur.2025.1524324, 39968458 PMC11834867

[13] Trinidade A Cabreira V Goebel JA Staab JP Kaski D Stone J. Predictors of persistent postural-perceptual dizziness (PPPD) and similar forms of chronic dizziness precipitated by peripheral vestibular disorders: a systematic review. J Neurol Neurosurg Psychiatry. (2023) 94:904–15. doi: 10.1136/jnnp-2022-330196 36941047

[14] Webster KE Harrington-Benton NA Judd O Kaski D Maarsingh OR MacKeith S . Pharmacological interventions for persistent postural-perceptual dizziness (PPPD). Cochrane Database Syst Rev. (2023) 3:CD015188. doi: 10.1002/14651858.CD015188.pub2, 36906836 PMC9997546

[15] Webster KE Harrington-Benton NA Judd O Kaski D Maarsingh OR MacKeith S . Non-pharmacological interventions for persistent postural-perceptual dizziness (PPPD). Cochrane Database Syst Rev. (2023) 3:CD015333. doi: 10.1002/14651858.CD015333.pub2, 36912784 PMC10011873

[16] Suica Z Behrendt F Ziller C Gäumann S Schädler S Hilfiker R . Comparative effectiveness of non-pharmacological treatments in patients with persistent postural-perceptual dizziness: a systematic review and effect sizes analyses. Front Neurol. (2024) 15:1426566. doi: 10.3389/fneur.2024.1426566, 39070052 PMC11272556

[17] Zang J Zheng M Chu H Yang X. Additional cognitive behavior therapy for persistent postural-perceptual dizziness: a meta-analysis. Braz J Otorhinolaryngol. (2024) 90:101393. doi: 10.1016/j.bjorl.2024.101393, 38350404 PMC10867767

[18] Zheng Y Guo Z Liu X Chen H Gang W Chen H . Effect of conservative therapy for persistent postural-perceptual dizziness: a systematic review and meta-analysis. Front Psychiatry. (2025) 16:1676218. doi: 10.3389/fpsyt.2025.1676218, 41244873 PMC12612630

[19] Piatti D De Angelis S Paolocci G Minnetti A Manzari L Verdecchia DH . The role of vestibular physical therapy in managing persistent postural-perceptual dizziness: a systematic review and meta-analysis. J Clin Med. (2025) 14:5524. doi: 10.3390/jcm14155524, 40807145 PMC12347945

[20] Li Y Pei X Ding R Liu Z Xu Y Wang Z . Effect of vestibular rehabilitation therapy in patients with persistent postural perceptual dizziness: a systematic review and meta-analysis. Front Neurol. (2025) 16:1599201. doi: 10.3389/fneur.2025.1599201, 41069431 PMC12504085

[21] Yagi C Morita Y Kitazawa M Nonomura Y Yamagishi T Ohshima S . A validated questionnaire to assess the severity of persistent postural-perceptual dizziness (PPPD): the Niigata PPPD questionnaire (NPQ). Otol Neurotol. (2019) 40:e747–52. doi: 10.1097/MAO.0000000000002325, 31219964 PMC6641087

[22] Murofushi T Nishimura K Tsubota M. Isolated otolith dysfunction in persistent postural-perceptual dizziness. Front Neurol. (2022) 13:872892. doi: 10.3389/fneur.2022.872892, 35481262 PMC9038172

[23] Van De Wyngaerde KM Lee MK Jacobson GP Pasupathy K Romero-Brufau S McCaslin DL. The component structure of the dizziness handicap inventory (DHI): a reappraisal. Otol Neurotol. (2019) 40:1217–23. doi: 10.1097/MAO.0000000000002365 31469793

[24] Iglebekk W Tjell C Borenstein P. Can the bending forward test be used to detect a diseased anterior semi-circular canal in patients with chronic vestibular multi-canalicular canalithiasis (BPPV)? Acta Otolaryngol. (2019) 139:1067–76. doi: 10.1080/00016489.2019.1667529 31588860

[25] Zhang DP Zhang YH Liu BY Zhang HL Zhao M. Differential diagnosis of orthostatic dizziness with persistent postural-perceptual dizziness and its underlying mechanisms. Front Neurol. (2025) 16:1642869. doi: 10.3389/fneur.2025.1642869 41103515 PMC12520903

[26] Chang T-P Schubert MC. Visual vertigo and motion sickness is different between persistent postural-perceptual dizziness and vestibular migraine. Am J Otolaryngol. (2024) 45:104321. doi: 10.1016/j.amjoto.2024.104321, 38696894

[27] Storm R Krause J Blüm S-K Wrobel V Frings A Helmchen C . Visual and vestibular motion perception in persistent postural-perceptual dizziness (PPPD). J Neurol. (2024) 271:3227–38. doi: 10.1007/s00415-024-12255-x, 38441610 PMC11136745

[28] Sereda A Lam JC Hazar AM Ellmers T Golding J Kaski D. Psychological variables mediate symptoms in persistent postural-perceptual dizziness (PPPD): a cross-sectional self-report study. Eur J Neurol. (2025) 32:e70339. doi: 10.1111/ene.70339, 40853180 PMC12376309

[29] Zhang L Jiang W Tang L Liu H Li F. Older patients with persistent postural-perceptual dizziness exhibit fewer emotional disorders and lower vertigo scores. Sci Rep. (2022) 12:11908. doi: 10.1038/s41598-022-15987-w, 35831350 PMC9279357

[30] Fukushima A Kabaya K Minakata T Katsumi S Esaki S Iwasaki S. Age-related differences in the characteristics of persistent postural-perceptual dizziness. Front Neurol. (2024) 15:1378206. doi: 10.3389/fneur.2024.1378206, 38708003 PMC11066216

[31] Habs M Strobl R Grill E Dieterich M Becker-Bense S. Primary or secondary chronic functional dizziness: does it make a difference? A DizzyReg study in 356 patients. J Neurol. (2020) 267:212–22. doi: 10.1007/s00415-020-10150-9, 32852579 PMC7718176

[32] Teh CS Prepageran N. The impact of disease duration in persistent postural-perceptual dizziness (PPPD) on the quality of life, dizziness handicap and mental health. J Vestib Res. (2022) 32:373–80. doi: 10.3233/VES-210087, 34924408

[33] Park JH Nguyen TT Kim SH Park JY Na S Jeon EJ . Clinical characteristics of persistent postural-perceptual dizziness and its visual subtype in Korean patients: a multicenter cross-sectional study. Brain Behav. (2024) 14:e3389. doi: 10.1002/brb3.3389, 38391108 PMC10831130

[34] Powell G Penacchio O Derry-Sumner H Rushton SK Rajenderkumar D Sumner P. Visual stress responses to static images are associated with symptoms of persistent postural perceptual dizziness (PPPD). J Vestib Res. (2022) 32:69–78. doi: 10.3233/VES-190578, 34151873

[35] Steensnaes MH Knapstad MK Goplen FK Berge JE. Persistent postural-perceptual dizziness (PPPD) and quality of life: a cross-sectional study. Eur Arch Otorrinolaringol. (2023) 280:5285–92. doi: 10.1007/s00405-023-08040-7, 37256345 PMC10620245

[36] Castillejos-Carrasco-Muñoz R Peinado-Rubia AB Lérida-Ortega MÁ Ibáñez-Vera AJ Tapia-Toca MC Lomas-Vega R. Validity and reliability of the Niigata PPPD questionnaire in a Western population. Eur Arch Otorrinolaringol. (2023) 280:5267–76. doi: 10.1007/s00405-023-08038-1, 37266755 PMC10620260

[37] Meletaki V Gobinet M Léonard J Elzière M Lopez C. French adaptation and validation of the Niigata PPPD questionnaire: measure of severity of persistent postural-perceptual dizziness and its association with psychiatric comorbidities and perceived handicap. Front Neurol. (2024) 15:1388805. doi: 10.3389/fneur.2024.1388805, 39139768 PMC11319117

[38] Kim H-J Park JH Kim J-S. Diagnostic utility of Niigata persistent postural-perceptual dizziness (PPPD) questionnaire (NPQ) for PPPD in comparison to dizziness handicap inventory. J Vestib Res. (2026) 36:154–63. doi: 10.1177/09574271251382800, 40986615

[39] Gerber E-Y Hilfiker R Wandel J Behrendt F Chételat S El Khadlaoui S . Confirmatory factor analysis and Rasch analysis of the German revised version of the Niigata PPPD questionnaire: NPQ-R. Front Neurol. (2026) 17:1797481. doi: 10.3389/fneur.2026.1797481, 42028211 PMC13099289

[40] Anagnostou E Armenis G Zachou A Storm R Sprenger A Helmchen C. The Athens–Lübeck questionnaire: a tool to discriminate between subtypes of persistent postural-perceptual dizziness. Front Neurol. (2025) 16:1550469. doi: 10.3389/fneur.2025.1550469, 40212608 PMC11983643

[41] De Vestel C De Hertogh W Van Rompaey V Vereeck L. Comparison of clinical balance and visual dependence tests in patients with chronic dizziness with and without persistent postural-perceptual dizziness: a cross-sectional study. Front Neurol. (2022) 13:880714. doi: 10.3389/fneur.2022.880714, 35685740 PMC9170888

[42] Aharoni MMH Lubetzky AV Arie L Krasovsky T. Factors associated with dynamic balance in people with persistent postural perceptual dizziness: a cross-sectional study using a virtual-reality four square step test. J Neuroeng Rehabil. (2021) 18:55. doi: 10.1186/s12984-021-00852-0 33766072 PMC7993529

[43] Özdemir HN Charlton J Cortese E Di Stadio A Herdman D Kaski D. Persistent postural-perceptual dizziness: a practical approach to diagnosis and patient communication. Eur J Neurol. (2026) 33:e70494. doi: 10.1111/ene.70494, 41657061 PMC12884132

[44] Axer H Finn S Wassermann A Guntinas-Lichius O Klingner CM Witte OW. Multimodal treatment of persistent postural-perceptual dizziness. Brain Behav. (2020) 10:e01864. doi: 10.1002/brb3.1864, 32989916 PMC7749543

[45] Yamato A Yagi C Kimura A Kai R Kitazawa M Yamagishi T . Is vestibular rehabilitation as effective for persistent postural-perceptual dizziness as for chronic unilateral vestibular hypofunction? Otol Neurotol. (2025) 46:170–5. doi: 10.1097/MAO.0000000000004397, 39663796

[46] Choi SY Choi JH Oh EH Oh SJ Choi KD. Effect of vestibular exercise and optokinetic stimulation using virtual reality in persistent postural-perceptual dizziness. Sci Rep. (2021) 11:14437. doi: 10.1038/s41598-021-93940-z, 34262120 PMC8280184

[47] Al-Omari MM Abuzaid SM Khair HJ Manaf H Alghwiri AA. The effect of using virtual reality on balance in people with persistent postural-perceptual dizziness: a randomized controlled trial. J Vestib Res. (2025) 35:213–24. doi: 10.1177/09574271251326587, 40085770

[48] Simanavicius V Mockeviciene D Lebedeva M Espírito Santo RCD Zaliene L Staskevicius A . Effect of vestibular rehabilitation therapy in PPPD: short-term results from a prospective observational study. J Clin Med. (2025) 14:7761. doi: 10.3390/jcm14217761, 41227157 PMC12608183

[49] Teh CSL Abdullah NA Kamaruddin NR Judi KBM Fadzilah I Zainun Z . Home-based vestibular rehabilitation: a feasible and effective therapy for persistent postural perceptual dizziness (a pilot study). Ann Otol Rhinol Laryngol. (2023) 132:566–77. doi: 10.1177/00034894221111408 35794811

[50] Law JHJ Koh HY Kua A. Optokinetic stimulation in the rehabilitation of visually induced dizziness in people with vestibular disorders: a systematic review. Clin Rehabil. (2024) 38:1001–22. doi: 10.1177/02692155241244932, 38584422

[51] Staab JP Ruckenstein MJ Amsterdam JD. A prospective trial of sertraline for chronic subjective dizziness. Laryngoscope. (2004) 114:1637–41. doi: 10.1097/00005537-200409000-00025 15475796

[52] Lubetzky AV Aharoni MMH Arie L Krasovsky T. People with persistent postural-perceptual dizziness demonstrate altered postural strategies in complex visual and cognitive environments. J Vestib Res. (2021) 31:505–17. doi: 10.3233/VES-201552, 33749625

[53] Yagi C Kimura A Kai R Yamagishi T Ohshima S Izumi S . Long-term outcomes of pharmacotherapy in patients with persistent postural-perceptual dizziness. Front Neurol. (2025) 16:1566898. doi: 10.3389/fneur.2025.1566898, 40177407 PMC11961416

[54] Moreno-Ajona D. Persistent postural-perceptual dizziness versus vestibular migraine: a narrative review. Headache. (2026) 66:298–306. doi: 10.1111/head.15078, 41147266 PMC12849528

[55] Herdman D Norton S Murdin L Frost K Pavlou M Moss-Morris R. The INVEST trial: a randomized feasibility trial of psychologically informed vestibular rehabilitation versus current gold standard physiotherapy for people with persistent postural perceptual dizziness. J Neurol. (2022) 269:4753–63. doi: 10.1007/s00415-022-11107-w, 35397754 PMC8994825

